# Proposing an Affordable Plasma Device for Polymer Surface Modification and Microbial Inactivation

**DOI:** 10.3390/molecules29174270

**Published:** 2024-09-09

**Authors:** William Chiappim, Felipe Vicente de Paula Kodaira, Gisele Fátima Soares de Castro, Diego Morais da Silva, Thayna Fernandes Tavares, Ana Carla de Paula Leite Almeida, Bruno Henrique Silva Leal, Antje Quade, Cristiane Yumi Koga-Ito, Konstantin Georgiev Kostov

**Affiliations:** 1 Laboratory of Plasmas and Applications, Department of Physics, School of Engineering and Sciences, São Paulo State University (UNESP), Guaratinguetá 12516-410, SP, Brazil; kodaira.felipe@gmail.com (F.V.d.P.K.); thayna.tavares@unesp.br (T.F.T.); ana.c.almeida@unesp.br (A.C.d.P.L.A.); silva.leal@unesp.br (B.H.S.L.); 2 Department of Environment Engineering and Sciences Applied to Oral Health Graduate Program, São José dos Campos Institute of Science and Technology, São Paulo State University (UNESP), São José dos Campos 12247-016, SP, Brazil; gisele.s.castro@unesp.br (G.F.S.d.C.); diego.m.silva@unesp.br (D.M.d.S.); cristiane.koga-ito@unesp.br (C.Y.K.-I.); 3 Groupe de Recherches sur l’Energétique des Milieux Ionisés (GREMI), UMR 7344, CNRS/Université d’Orléans, 45067 Orléans, France; 4 Leibniz Institute for Plasma Science and Technology—INP, 17489 Greifswald, Germany; quade@inp-greifswald.de; 5 Oral Biopathology Graduate Program, São José dos Campos Institute of Science & Technology, São Paulo State University (UNESP), São José dos Campos 12245-000, SP, Brazil

**Keywords:** cold atmospheric plasma, dielectric barrier discharge, polyethylene, polymer surface modification, *Staphylococcus aureus*, *Candida albicans*, Vero cell, cytotoxicity, antimicrobial effects, affordable cost

## Abstract

This study proposes an affordable plasma device that utilizes a parallel-plate dielectric barrier discharge geometry with a metallic mesh electrode, featuring a straightforward 3D-printed design. Powered by a high-voltage supply adapted from a cosmetic plasma device, it operates on atmospheric air, eliminating the need for gas flux. Surface modification of polyethylene treated with this device was characterized and showed that the elemental composition after 15 min of plasma treatment decreased the amount of C to ~80 at% due to the insertion of O (~15 at%). Tested against *Candida albicans* and *Staphylococcus aureus*, the device achieved a reduction of over 99% in microbial load with exposure times ranging from 1 to 10 min. Simultaneously, the Vero cell viability remained consistently high, namely between 91% and 96% across exposure times. These results highlight this device’s potential for the surface modification of materials and various infection-related applications, boasting affordability and facilitating effective antimicrobial interventions.

## 1. Introduction

Surface modification by non-thermal atmospheric pressure plasma (NTAPP) is a well-established technique [1,2]. However, developing and optimizing NTAPP devices is essential, as there is much to be improved. NTAPP devices present a range of advantages over other industrial chemical processes, with the highlight being that they do not necessitate the use of solvents that are potentially dangerous for the environment, making this technology green [3]. Another point to highlight is their versatility in modifying sensitive surfaces without changing the bulk properties [4,5].

On the other hand, microbial contamination represents a pervasive and significant concern across various surfaces, ranging from healthcare facilities to industrial settings and everyday life [6,7]. Microorganisms, including bacteria and fungi, can increase rapidly, posing risks to human health, product integrity, and the overall well-being of ecosystems [8]. In the healthcare system, nosocomial infections, also known as healthcare-associated infections (HAIs), are a prominent consequence of microbial contamination. These infections can be caused by bacteria such as *Staphylococcus aureus*, emphasizing the critical importance of stringent hygiene measures and infection control protocols [9]. In industrial contexts, surface contamination by microorganisms can compromise the quality and safety of products, leading to financial losses and potential harm to consumers [10,11,12]. In the food industry, for instance, pathogenic bacteria like *Salmonella* and *Escherichia coli* can cause foodborne illnesses, prompting the need for rigorous sanitation practices [13,14]. Beyond specific sectors, the ubiquitous nature of microorganisms means that contamination is an ever-present challenge in our surroundings. The advent of global travel and increased interconnectedness has facilitated the spread of infectious agents, making managing and preventing contamination an international public health priority [15]. Understanding the background of contamination by microbes is crucial for developing effective strategies to mitigate risks, whether in healthcare, industry, or daily life. Robust hygiene practices, proper sanitation, and the development of advanced technologies for microbial control are essential components of a comprehensive approach to addressing the challenges posed by microbial contamination [16,17].

Therefore, a device that subtly alters the surface of a material without affecting its bulk properties and inactivates microorganisms is essential for preventing microbial contamination while preserving the integrity of materials. In this context, NTAPP technology emerges as an alternative tool for germ control. NTAPP refers to a unique state of matter consisting of partially ionized gasses, often generated at or near room temperature and pressure, without reaching the high temperatures associated with traditional plasma [18,19,20,21,22,23]. Unlike more conventional hot plasmas, created at high temperatures and requiring specialized equipment, NTAPP operates at ambient temperatures, making it suitable for a wide range of applications, including medicine, materials science, and food safety [24,25,26,27]. One of the distinctive features of NTAPP is its ability to produce a complex mixture of diverse reactive species, including ions, electrons, reactive oxygen and nitrogen species (RONS), and radicals, without significantly increasing the temperature of the surrounding environment [28,29,30,31,32]. This makes NTAPP a promising technology for applications that require targeted and controlled delivery of reactive species, such as in medical treatments, surface modifications, and decontamination processes [33]. NTAPP has gained attention in medicine due to its great potential in wound healing, cancer treatment, and microbial disinfection [34,35,36,37,38,39,40,41]. The reactive species generated by NTAPP can interact with biological tissues in a controlled manner, offering potential benefits for various therapeutic applications [42,43,44]. In materials science, NTAPP is utilized for modifying the surfaces of materials, enhancing their adhesion, wettability, and other surface properties [45,46]. NTAPP has shown promising results in food safety as a non-thermal method for microbial decontamination [47,48,49,50,51,52,53,54]. It can be applied to fruits, vegetables, and other food products to reduce pathogens and extend shelf life without compromising the sensory and nutritional qualities of the food. The versatility, efficiency, and non-thermal nature of cold atmospheric plasma make it an exciting area of research and application, potentially revolutionizing various industries by offering innovative and sustainable solutions to longstanding challenges.

As a significant source of NTAPP, the utilization of dielectric barrier discharge (DBD) has emerged as a promising method for decontamination in many fields due to its efficacy, versatility, and ability to operate at atmospheric pressure [55,56]. DBD involves the generation of a non-thermal plasma between two electrodes separated by a dielectric barrier. This discharge creates a unique environment rich in reactive species such as ozone, UV radiation, reactive oxygen, and nitrogen, contributing to its powerful decontamination capabilities [57]. DBD has effectively sterilized medical instruments, surfaces, and even air in healthcare. Its ability to target a broad spectrum of microorganisms, including bacteria, viruses, and fungi, makes it a valuable tool for infection control in hospitals and healthcare facilities [58,59,60,61]. DBD technology has also found applications in food safety. By subjecting food surfaces to DBD-generated plasma, microbial contaminants can be reduced or eliminated without compromising the quality of the food [48]. This non-thermal decontamination method offers an alternative to traditional heat-based processes, preserving the nutritional and sensory attributes of the treated food products [48]. The versatility of DBD extends to environmental applications, where it has been explored for air and water purification. DBD can eliminate airborne pathogens and pollutants in air treatment, improving indoor air quality [62]. DBD has shown capability in degrading pollutants and disinfecting water sources in water treatment.

Moreover, DBD’s ability to operate at atmospheric pressure without requiring a vacuum chamber makes it practical for various settings [63,64]. Its energy efficiency and minimal environmental impact further enhance DBD’s attractiveness for decontamination. The use of DBD technology represents a cutting-edge and versatile decontaminating approach in diverse fields. Its ability to target a wide range of contaminants while maintaining operational practicality positions DBD as a valuable tool in advancing industry sanitation and safety standards.

This work’s affordable plasma device (APD) incorporates a parallel-plate dielectric barrier discharge (PP-DBD) reactor with a metallic mesh. It uses 3D-printing technology to craft the other parts of the device. Three-dimensional printing offers several advantages, as follows. (i) Cost-effectiveness: Engineered with a focus on affordability, our APD leverages 3D printing for reactor construction, reducing the production costs. This cost-effective design makes the technology accessible for a broader range of applications and users. (ii) Versatile design: Using a parallel-plate configuration with a metallic mesh enhances the versatility of this APD. This design allows for the efficient and targeted delivery of plasma species, making it adaptable for various applications, including surface modification and antimicrobial interventions. (iii) Easy power supply adaptation: Incorporating simple high-voltage sources adapted from a commercial plasma system adds to the device’s advantages. This adaptation facilitates power delivery and draws from established technologies, contributing to the device’s reliability and scalability. (iv) Atmospheric air operation: The use of air as the working gas distinguishes this APD from other plasma jet devices. This feature eliminates the need for a gas flux, simplifying the operational requirements and making the device more user-friendly.

Therefore, the present work contributes to developing affordable plasma devices of the DBD type. This technology’s potential impact extends across healthcare, industrial, and environmental settings, showcasing its significance in diverse applications.

## 2. Results and Discussion

This section is divided into three parts. The first part characterizes the APD, focusing on electrical properties, spectroscopic emissions, and thermal parameters. It uses OES to identify excited species and radicals, which assists our understanding of plasma’s interactions with materials and its biomedical potential. The second part explores the APD’s application in modifying polyethylene (PE) surfaces and analyzing changes through WCA measurements, FTIR, and XPS. These techniques assess hydrophilicity, chemical functional groups, and elemental composition, revealing enhancements in surface properties. The final part presents a discussion on the antimicrobial activity and cytotoxicity tests conducted on the plasma-treated surfaces. These tests assessed microbial inhibition and biocompatibility using standardized methods and the MTT assay, determining the treated materials’ safety and potential biomedical applications. Overall, this study provides a detailed understanding of this APD’s properties, demonstrates its effectiveness in surface modification, and evaluates its biological effects, paving the way for future innovations in materials science and biomedicine.

### 2.1. Characterization of the Affordable Plasma Device

#### 2.1.1. Electrical Characterization

**Figure 1:**
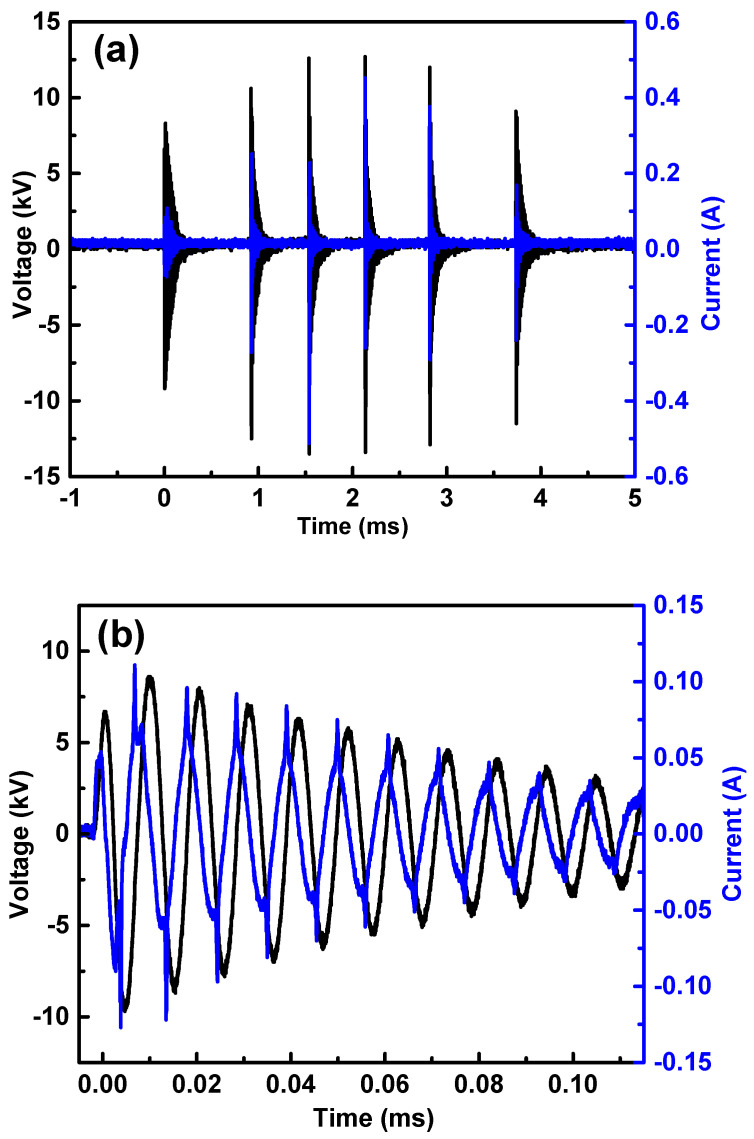
(**a**) Voltage and current signals of a 6-burst package; (**b**) detailed view of voltage and current signals into the first burst, wherein the characteristic sharp DBD signals can be observed in the current signal.

As indicated in Figure 9 (Section Materials and Methods), the applied voltage signal is characterized by six sinewave packages repeated at a frequency of 60 Hz. In Figure 1a, the voltage and current signals within one of these burst packages are illustrated. Notably, the voltage signals exhibit varying amplitudes, with the middle ones reaching higher values. However, the current signals reveal consistent discharge across all pulses. In Figure 1b, a closer examination of the signals in the first burst (Figure 1a) is presented. This magnified view allows a more detailed observation of the current behavior in a dielectric barrier discharge (DBD). It becomes apparent that the current signal consists of a sequence of sharp peaks superimposed on a large displacement current. This sinewave is phase-shifted in relation to the voltage signal. The current peaks precede the voltage peaks and always occur at the rising or falling front of the voltage. These observations collectively indicate the formation of a filamentary discharge [65]. The discharge power determined with the Lissajous figure method using voltage-versus-charge calculations revealed that a discharge power value of 3.5 ± 0.2 W was taken for this six-pulse package [66].

#### 2.1.2. Spectroscopic Emissions and Thermal Parameters

**Figure 2:**
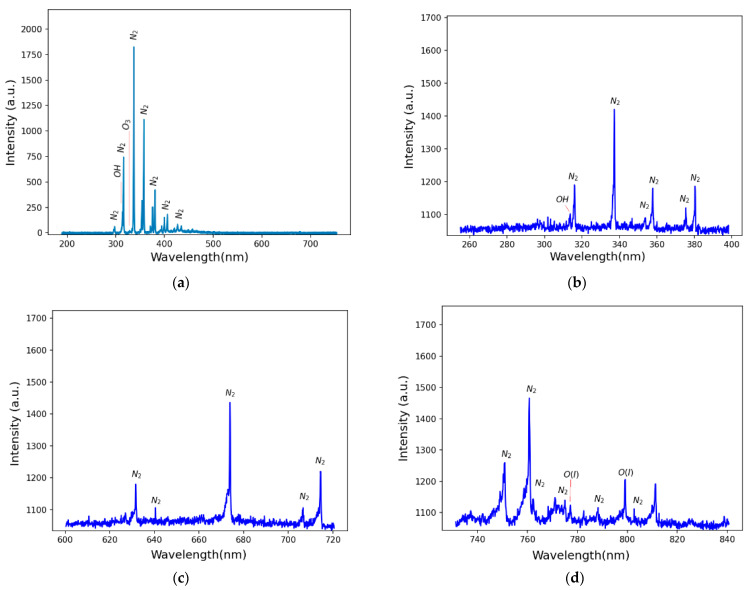
Optical emission spectrum: (**a**) the full spectrum from 200 to 750 nm; (**b**–**d**) more detailed spectra of N_2_ species for specific regions, namely 260–400 nm, 600–720 nm, and 730–840 nm, respectively.

The optical emission spectrum presented in Figure 2a illustrates the results obtained with a power of 3.5 W applied to the APD using an Avantes optical emission spectrometer operated in the UV–visible range of 200–750 nm. The spectrum reveals prominent emissions from N_2_ lines ($C^{3}\Pi_{u}\to B^{3}\Pi_{g}$) in the second positive system (SPS), featuring various vibrational transitions (Δv = v′ − v″) and emission bands at 297 nm (Δv = +2), 313 nm (Δv = +1), 315 nm (Δv = +1), 337 nm (Δv = 0), 357 nm (Δv = −1), 380 nm (Δv = −2), 406 nm (Δv = −3), 420 nm (Δv = −4), 426 nm (Δv = −4), and 434 nm (Δv = −4). Additionally, there are emissions from OH radicals (Σ2→Π2) at around 309 nm and O_3_ at 330 nm. While the presented spectroscopy pertains to a power of 4.5 W, it is noteworthy that reducing the reactor power maintains the same species, albeit with reduced intensity in the emission peaks. Further spectroscopic analyses were performed in specific regions using the Horiba spectrometer (730–840 nm), and these results are delineated in Figure 2b–d. Figure 2c,d specifically showcase emissions of N_2_ lines ($B^{3}\prod \to A^{3}\sum$) in the first positive system (FPS), with bands at 639 nm (Δv = +3), 670 nm (Δv = +3), and 804 nm (Δv = +1). Additionally, they depict the second emissions of N_2_ lines ($C^{3}\Pi_{u}\to B^{3}\Pi_{g}$) in the second positive system (SPS) at 632 nm (Δv = +1), 706 nm (Δv = −1), 716 nm (Δv = −1), 750 nm (Δv = +2), 762 nm (Δv = +2), 775 nm (Δv = +2), 789 nm (Δv = +3), 799 nm (Δv = +3), and 812 nm (Δv = +3). Additionally, emissions from oxygen were identified in O I (transition: 3p^5^ P → 3s^5^ S^0^) at 777 nm. These results collectively provide valuable insights into the composition and behavior of the atmospheric-pressure plasma discharge conditions.

**Figure 3:**
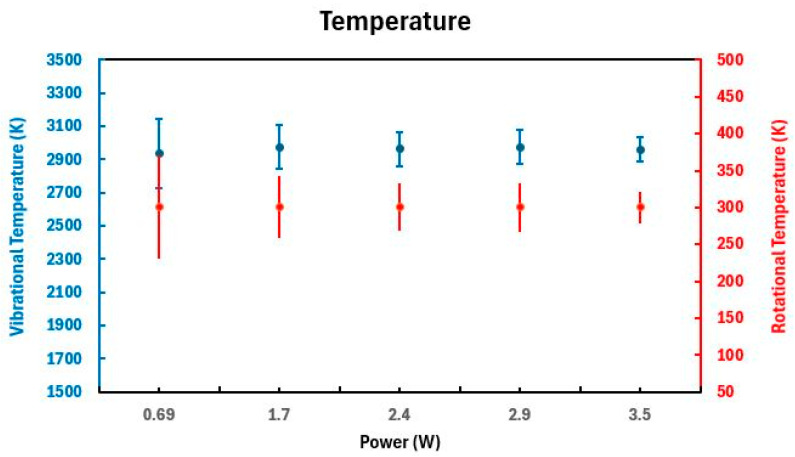
N_2_ rotational and vibrational temperatures for different discharge powers.

The experimental spectra acquired under diverse experimental configurations were subjected to adjustment by applying the MassiveOES GUI 1.0 software. Engineered for this purpose, the software aligns spectral simulations stored within its database with the experimentally obtained data. This process facilitates the extraction of diatomic-molecule vibrational and rotational temperatures, as Voráč et al. outlined [67]. The wavelength range of the molecular nitrogen emission selected for temperature analyses in this investigation spans 360 to 383 nm and represents the N_2_ emission lines from the second positive system. The outcomes of these analyses as a function of the discharge power are graphically depicted in Figure 3. Conventionally, determining the gas temperature stems from examining the system’s rotational structure (T_rot_). The second positive system of N_2_ is characterized by low energies for rotational excitation and abbreviated transition times [68], fostering equilibrium between molecules in rotational states and gas molecules. The vibrational temperature (T_vib_) elucidates the vibration modes of molecules and correlates with the pace of chemical reactions. Elevated T_vib_ values amplify the likelihood of chemical reactions between the plasma and the target, as elucidated by Nascimento et al. [69]. Under the scrutinized experimental conditions, observations reveal a sustained rotational temperature of 300 ± 32 K (approximately 27 °C) within the reactor. Concurrently, the average vibrational temperature persists at 2961 ± 120 K, demonstrating resilience to variations in discharge power.

### 2.2. Polyethylene Surface Modification

#### 2.2.1. Characterization via Water Contact Angle and Fourier-Transform Infrared Spectroscopy

**Figure 4:**
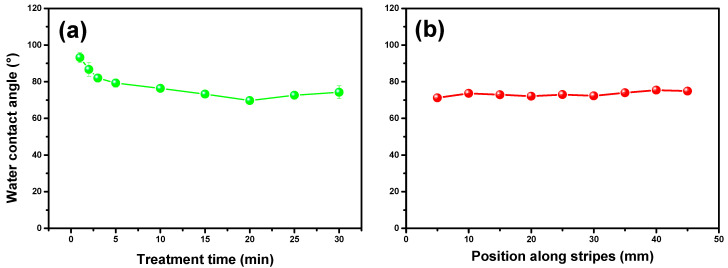
(**a**) Mean values of water contact angle for polyethylene samples measured after APD treatment for between 1 and 30 min; (**b**) mean values of water contact angle measurements taken along the central axis of the samples at 5.0 mm intervals during 15 min of treatment.

Figure 4 illustrates the WCA measurements for the polyethylene (PE) samples. The WCA of the untreated substrate was recorded at 95.1°. Figure 4a shows the average WCA values for each treatment time, with measurements taken along the central axis of each sample at 5.0 mm intervals. The samples of 40.0 × 10.0 mm were placed with one edge at the border of the device and the other edge at the center; this placement was chosen to evaluate the homogeneity of the treatment. Nine samples were measured for each treatment duration, providing an average value with corresponding error bars. This systematic approach ensures a reliable representation of the WCA across different treatment times. Figure 4b shows the WCA measurements taken along the central axis for a 15 min treatment time. These measurements reveal the uniformity of the plasma treatment on the PE surface, indicating consistent modification across the sample. Therefore, the plasma treatment resulted in a notable reduction in the WCA, with values ranging between 76 and 93 degrees depending on the treatment duration. This reduction signifies an increase in the hydrophilicity of the PE surface. The reduction in WCA is attributed to the formation of oxygen-containing functionalities on the polymer surface. According to the literature [70,71], dielectric barrier discharge plasma treatments induce the formation of functional groups such as C–O, O–C=O and C=O on polymer surfaces. This chemical modification enhances the surface energy, leading to a decreased WCA. These studies corroborate the findings presented here, demonstrating the efficacy of plasma treatments in modifying polymer surfaces to improve their wettability.

Thus, the data presented in Figure 4 underscore the effectiveness of our APD in altering the surface properties of PE, making it more hydrophilic. The uniform reduction in WCA across the treated samples highlights the consistency and reliability of the plasma treatment process. This modification is crucial for applications requiring enhanced surface wettability and can be further explored for its potential in various industrial and biomedical applications.

**Figure 5:**
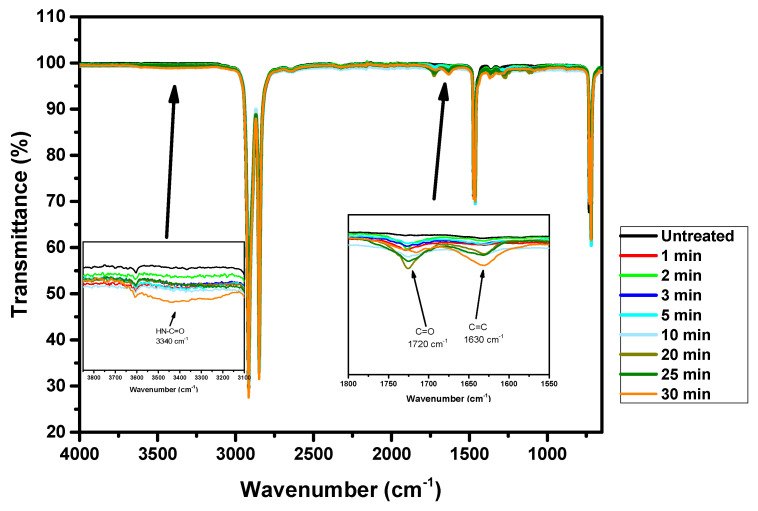
Full FTIR spectra of untreated polyethylene samples and polyethylene samples treated for between 1 and 30 min. The insets highlight the main changes observed after plasma treatment.

Figure 5 presents the Fourier-transform infrared spectra of PE samples treated with the APD for durations ranging from 1 to 30 min alongside the spectrum of the untreated PE sample. The FTIR technique probes depths ranging from 0.5 to 5.0 µm, making it an excellent tool for detecting and analyzing chemical changes on the surface and in the inner layers of polyethylene samples. By comparing the spectra of treated and untreated samples, it is possible to identify the introduction and alteration of functional groups within the polymer matrix. The FTIR spectrum of the untreated PE sample exhibits characteristic absorption bands associated with the polymer’s intrinsic chemical structure, primarily consisting of CH_2_ asymmetric stretching vibrations around 2915 cm^−1^, CH_2_ symmetric stretching vibrations at 2848 cm^−1^, and bending deformation and rocking deformation vibrations around 1470 cm^−1^ and 730 cm^−1^, respectively [72]. The spectra of PE samples treated with the APD for varying durations reveal significant changes, indicating the incorporation of new functional groups and chemical modifications. Peaks corresponding to oxygen-containing functionalities such as C=O (carbonyl, ~1720 and 1680 cm^−1^) and HN-C=O (hydroxyl, broad peak around ~3340 cm^−1^) become more pronounced with increasing treatment time [2,73]. These changes are consistent with oxidative processes induced by the plasma treatment. The depth penetration capability of FTIR confirms that the chemical modifications induced by this APD are not limited to the surface but extend into the near-surface layers of the PE samples [5]. This is crucial for applications requiring durable and robust surface properties. Introducing oxygen-containing groups can significantly enhance the functional properties of PE, such as its adhesion, wettability, and compatibility with other materials. These modifications make plasma-treated PE suitable for various applications, including biomedical devices, packaging, and surface coatings, sparking interest in the potential uses of this research. Figure 5 demonstrates the efficacy of APD treatment in chemically modifying PE at both the surface level and the near-surface level. The FTIR spectra provide clear evidence of the introduction of new functional groups, which can enhance the material’s performance in various applications. The ability to tailor a material’s chemical composition through applying plasma treatment and controlling the treatment duration opens up new possibilities for using PE in advanced technological and industrial fields.

Figure 5 displays a peak at 1720 cm^−1^ related to C=O groups, whose intensity increases with the treatment time. A new band is also observed at approximately 1680 cm^−1^, attributed to the C=O stretching groups in amides [73]. However, the band at approximately 3340 cm^−1^ plays a crucial role. This band can be associated with stretching in the HN-C=O group [74]. The peak at 1630 cm^−1^ also increases with the treatment time; this peak can be attributed to C=C bands, meaning it may arise due to the formation of double bonds as a result of plasma-induced dehydrogenation or crosslinking during the treatment [75]. This confirmation is a significant step in our research, as it validates the presence of nitrogen in the plasma-treated polymers.

Figure 5 shows the increase in the intensity of these bands (1720, 1680, and 3340 cm^−1^) with the APD treatment. The intensity of the C=O groups rises as the APD exposure period extends, and a similar trend is observed for the C=O stretching groups in amides and the HN-C=O stretching group. The FTIR spectra demonstrate that plasma treatment on polymers often involves incorporating oxygen and nitrogen atoms into the surface [76].

These changes in the FTIR spectra indicate significant chemical modifications on the polymer surface due to the plasma treatment. The increased intensity of the bands at 1720 cm^−1^ suggests the formation of additional functional groups that can serve as binding sites for other molecules. The presence of a band at 3340 cm^−1^ reflects the incorporation of nitrogen, which may affect the chemical and physical properties of the treated polymer. Such modifications can enhance the material’s adhesive properties and compatibility with other materials, making plasma-treated polymers more versatile for various applications and potentially revolutionizing the field of materials science and polymer chemistry.

#### 2.2.2. X-ray Photoelectron Spectroscopy

**Figure 6:**
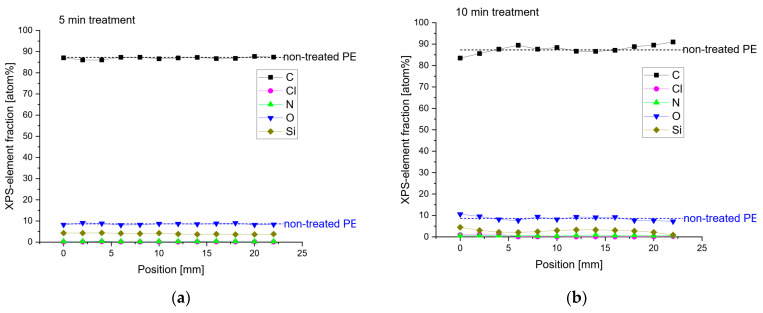

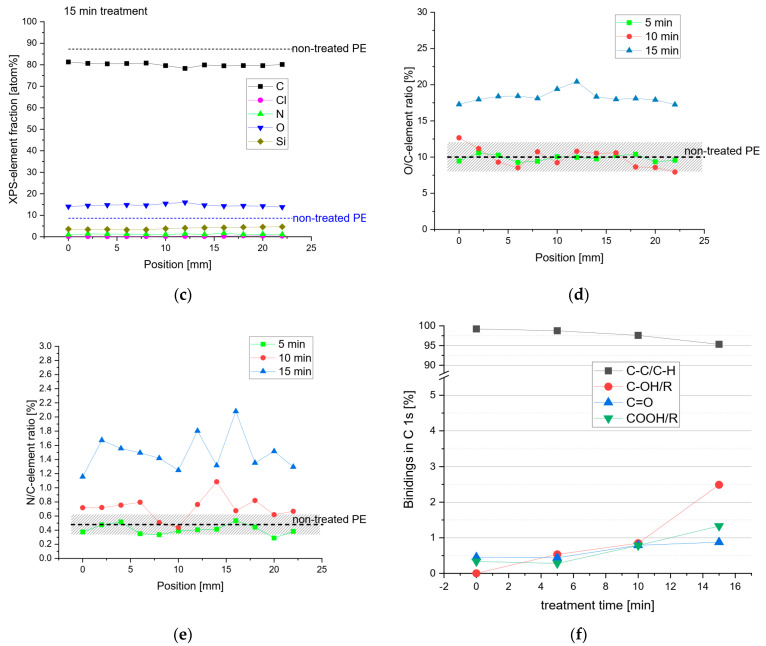
XPS analysis of polyethylene before and after treatment with the affordable plasma device, where (**a**–**c**) show the elemental fractions in the samples after 5, 10, and 15 min of APD treatment, (**d**,**e**) display the O/C and N/C ratios after 5, 10, and 15 min of APD treatment, and (**f**) shows the C1s binding energies.

Figure 6 shows the results of the XPS analysis used to study the degree of chemical modifications on the surface of substrates within a depth range of 5.0 to 10.0 nm. This technique evaluates surface oxidation and the percentage of atomic species on the PE surface. For this purpose, the sum of C, O, N, and Si was assumed to be 100 at%, meaning that the proportion of H in the calculations was neglected. The untreated PE reference contains 87 at% C, 9 at% O, 4 at% Si, and traces of N and Cl, indicating significant contamination by O, Si, Cl, and N from unknown sources. The O/C ratio of this reference material is 10%, and the N/C ratio is 0.5%. Figure 6a–c show the elemental fractions in samples after 5, 10, and 15 min of APD treatment, respectively. Compared to the untreated PE, the elemental composition of PE after 5 min of plasma treatment did not change significantly. After 10 min of plasma treatment, the elemental composition changed only slightly.

However, after 15 min of plasma treatment, the amount of C decreased to 80 at% due to the insertion of O, which increased to 15 at%. Figure 6d,e display the O/C and N/C ratios, respectively, with the shaded areas representing the standard deviation of these elemental ratios. Only the 15 min plasma treatment significantly increased the O/C ratio compared to the untreated and short-term treated PE.

The N/C ratio of the long-term-treated PE rose from 0.5% to 1.6% compared to the untreated PE sample. Figure 6f shows the C1s binding energies. Due to the plasma treatment, the insertion of O-containing functional groups can be observed. The longer the plasma treatment was applied, the more O-containing functional groups were recognizable.

This corroborates the data found in the FTIR spectra. The results indicate that the APD treatment added O atoms and a small amount of N, with only trace amounts of Si atoms on the PE surface.

### 2.3. Cytotoxicity and Antimicrobial Activity

**Figure 7:**
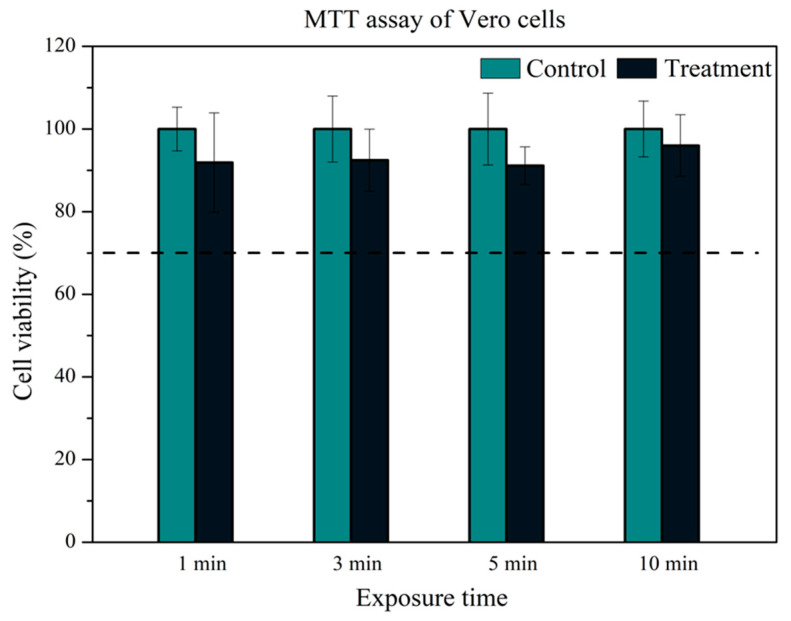
Outcomes from the MTT assay conducted on Vero cells. This graph depicts the cell viability (%) following exposure to plasma for 1, 3, 5, and 10 min, along with the corresponding control groups. The dashed line denotes the threshold of 70% cell viability.

All experimental groups treated with the APD demonstrated viability levels exceeding 70%, indicating that treatments administered within 1 to 10 min are non-cytotoxic (Figure 7). Specifically, the cell viability for treatment durations of 1, 3, 5, and 10 min was recorded as 91.88 ± 12.02%, 92.44 ± 7.53%, 91.12 ± 4.56%, and 96.00 ± 7.46%, respectively. These results are consistent with those of Gerber et al. [77], who studied the exposure of the Vero cell line to a low-temperature air DBD plasma treatment. In their study, Vero cells plated inside a 24-well plate were exposed to the plasma for 10 min, and cell viability was assessed after 24 h. The viable cell percentage was above 80%, which is similar to the findings of this study. In the same survey by Gerber et al. [77], HeLa cells were exposed to the same plasma treatment under identical conditions.

However, the HeLa cell line exhibited a cell viability percentage below 70%, with values dropping below 20%, which is considered cytotoxic. According to the authors, the difference in the response between the two cell lines to the same DBD plasma treatment can be attributed to the cytophysiology of each cell line. The reactive species produced by plasma can play a crucial role in this behavior, potentially triggering different pathways that may lead to cell apoptosis. Borges et al. [78] also reported similar results by exposing Vero cells to a plasma jet treatment. In their study, cell viability after 24 h was above 80%, in contrast to the reduction they observed in the CFU count of *C. albicans*. The authors discussed that the difference in their results between fungal and mammalian cells could be attributed to differences in the protocols for antimicrobial activity tests and mammalian cell viability, leading to different concentrations and effects of reactive species on each cell type.

These studies collectively underscore the importance of understanding the specific responses of various cell types to plasma treatments. While Vero cells and other mammalian cells demonstrate high viability and non-cytotoxic responses to APD treatments, different cell types, such as HeLa cells, may respond differently due to their unique cytophysiological characteristics. This underscores the potential for plasma treatments to be tailored for specific applications, ensuring safety and efficacy across various biological contexts and highlighting the adaptability and versatility of our research in the field of biotechnology.

In our experiment, the control group representing 100% cell viability was established using a culture medium. This was chosen because it reliably provides a condition to confirm the cells’ maximum viability. The graph above does not depict this control group, as it was used solely as a reference benchmark. The other control groups were exposed only to PBS (phosphate-buffered saline) for the same duration as each plasma treatment. This strategy enabled us to assess whether any reduction in cell viability was due to prolonged exposure to PBS, thereby isolating the specific effects of plasma treatment on cell viability. Therefore, these controls, corresponding to each exposure time, are referred to as ‘treatment controls’. By employing these distinct control conditions, we were able to evaluate the impact of plasma treatment on cell viability more accurately while mitigating potential variables introduced by the experimental setup.

Table 1 presents the percentage reduction in *C. albicans* and *S. aureus* subjected to plasma treatments lasting 1, 3, 5, and 10 min.

The APD demonstrated remarkable efficacy in reducing *C. albicans*, reinforcing the earlier findings of Maisch et al. [79]. In this study, an impressive elimination of 99.9% of viable *Candida* cells was observed within a brief 40 s period of plasma treatment on planktonic cells. Furthermore, applying plasma to *Candida* biofilms resulted in the successful inactivation of the cells, with efficacy increasing proportionally with treatment time, achieving an elimination of over 99.9% after 7 min. Although a fungicidal effect was not observed in both cases, the derived conclusion from these findings is that the generated plasma agents exhibit synergistic effects, leading to a faster inactivation of pathogens. The DBD technique has also been widely reported as an efficient method for bacterial inhibition. Similarly, Khosravi et al. [80] significantly inhibited *S. aureus* and *E. coli*. Their inhibition results stemmed from the plasma’s capability of generating high amounts of reactive oxygen species (ROS), such as O_3_ and OH, as observed with optical emission spectroscopy [81]. The underlying mechanism for the antimicrobial action of DBD plasma involves the generation of various reactive species, including reactive oxygen and nitrogen species (RONS), which are highly effective in damaging microbial cells. These species can cause oxidative stress, leading to lipid peroxidation, protein denaturation, and DNA damage in microbial cells, ultimately resulting in cell death [82]. The efficacy of DBD plasma in treating both planktonic cells and biofilms is particularly significant, given the increased resistance of biofilms to conventional antimicrobial treatments. Moreover, the application of DBD plasma extends beyond fungus and bacteria. Studies have shown its potential in deactivating a broad spectrum of pathogens, including viruses and spores, making it a versatile tool in disinfection and sterilization processes. The ability of DBD plasma to achieve high levels of microbial inactivation within short treatment times highlights its potential for practical applications in medical device sterilization, wound care, and surface disinfection.

Numerous DBD reactors have been utilized in the literature for antimicrobial treatments. However, comparing these reactors is challenging due to the significant variation in operational parameters across different studies. For instance, Poiata et al. [83] employed a helium plasma generated by an asymmetric DBD device at atmospheric pressure, with parameters including a gas flow of 0.15 L/min, an applied voltage of 9 kV (peak to peak), and a frequency of 1.6 kHz, specifically for microorganism sterilization. On the other hand, Miranda et al. [84] used a coaxial DBD reactor with various gases, including compressed air (AC), helium (He), and argon (Ar), to assess its antimicrobial efficacy. In the present study, a planar DBD reactor operating at atmospheric pressure is utilized without the addition of external gas flow and with ambient air as the working gas. Despite these differences in reactor design and operational parameters, a commonality across all studies is the generation of a substantial amount of RONS, which play a crucial role in the inactivation of microorganisms. Given the versatility and effectiveness of DBD technology in producing RONS, its application is particularly noteworthy in the context of inactivating pathogenic organisms such as *C. albicans* and *S. aureus*. This underscores the potential of DBD reactors as a powerful tool in antimicrobial treatments, with broad implications for healthcare and sterilization processes.

In conclusion, various studies’ findings underscore DBD plasma’s potential as a powerful antimicrobial tool. Its rapid and effective action against a range of pathogens, coupled with its ability to generate reactive species, positions it as a promising technology for enhancing infection control and improving public health outcomes.

## 3. Materials and Methods

### 3.1. Affordable Plasma Device

**Figure 8:**
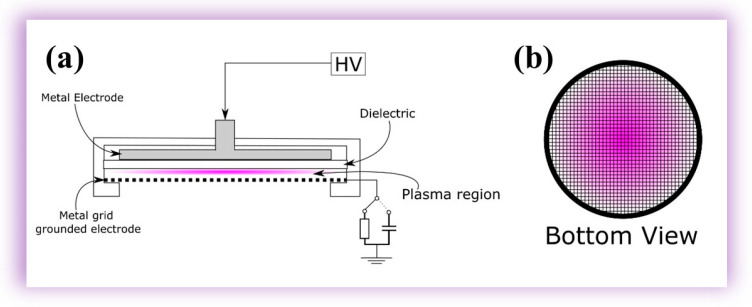
(**a**) DBD system schematic; (**b**) bottom-view illustration of the device in operation.

The APD utilized in this research integrates a parallel-plate dielectric barrier discharge (PP-DBD) reactor. It incorporates a stainless-steel mesh with a diameter of 80 mm and cells of 0.5 × 0.5 mm as a grounded electrode and a high-voltage aluminum electrode (HVAE) in the form of a disk with a diameter of 65 mm. The dielectric barrier is a 1 mm thick polyethylene (PE) slab with a diameter of 85 mm, completely covering the high-voltage electrode. The use of 3D-printing technology in the device’s construction is significant, as it allows for the precise and customized fabrication of the plastic parts that constitute the device. These parts were meticulously crafted with a 3D printer (Creality Ender 3 32 Bits, Creality, Shenzhen, China) using Polylactic Acid (PLA) filaments. The HVAE is linked to the high-voltage power supply, adapted from a commercial plasma system devised for cosmetic applications (Beauty Face, HTM, Amparo, SP, Brazil). This adaptation not only streamlines power delivery but also leverages established technologies, enhancing the reliability, portability, and scalability of the APD. Operable with atmospheric air, the APD eliminates the necessity for an air flux, streamlining operational requirements and enhancing user-friendliness at an affordable cost. The effective area of this device encompasses the entire area of the mesh electrode. Figure 8 schematically illustrates the discharge generated within a 1 mm gap between the dielectric barrier and the mesh electrode. The assembly of these components, including the electrodes and the dielectric barrier, is encased in a carefully designed cylindrical 3D-printed enclosure. Notably, the latter features an opening aligned with the mesh electrode so that the generated reactive species can exit the DBD reactor. Electrical characterization involves acquiring the applied voltage signal using an HV probe (P6015A, Tektronix, Beaverton, OR, USA) directly connected to the power supply. Discharge current and transferred charge waveforms are obtained by grounding the mesh electrode through a 100 Ω resistor or a 10 nF capacitor, respectively. In this study, optical emission spectroscopy (OES) was employed to characterize the primary plasma species within the UV–visible range of 200–750 nm. For this purpose, an Avantes optical emission spectrometer (AvaSpec-ULS2048X64T, Avantes, Apeldoorn, the Netherlands) with a spectral resolution (FWHM) of 0.384 nm was utilized.

Further refinement in spectroscopic measurements within the 730–840 nm range was achieved using a Horiba multi-channel spectrometer (model MicroHR, Horiba, Jundiaí, SP, Brazil) with an FWHM of 0.42 nm. In both spectrometers, the emitted light from the plasma jet was collected through optical fibers parallel to the mesh electrode. These optical fibers, featuring numerical apertures (NA) of 0.22, differed only in the diameters of their cores—1000 μm for the one connected to the Avantes spectrometer and 100 μm for the one connected to the Horiba spectrometer. The distance between the optical fiber and the mesh electrode was maintained at 4 cm. The acquisition of the optical spectrum was conducted using Avasoft software (Avantes Software) and HORIBA Scientific SynerJY (Horiba software version 1.0), ensuring a precise and detailed analysis of the emitted wavelengths. Figure 8a presents a comprehensive schematic configuration of the system, while Figure 1b provides a bottom view, showcasing the plasma through the mesh electrode. This experimental setup, designed to enable a thorough analysis of plasma behavior under controlled conditions, ensures the highest research quality and thoroughness.

**Figure 9:**
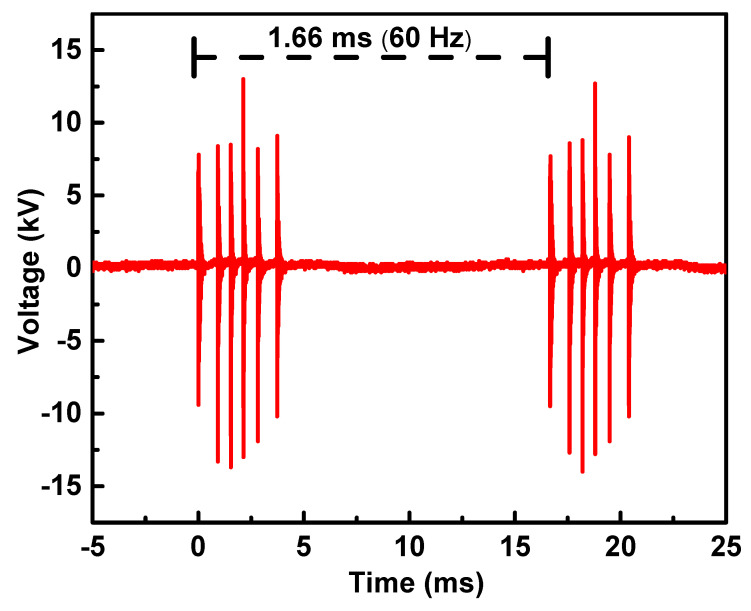
Discharge voltage waveforms of the APD operating in atmospheric air.

The selection of the mesh electrode was deliberate. It aimed to enhance the diffusion of species and radiation originating from the plasma region toward the external space, fostering interaction with neighboring surfaces. A simple power supply was employed, operating with a voltage signal featuring six bursts of high-voltage oscillations with a repetition frequency of 60 Hz and an amplitude of up to 15 kV, as depicted in Figure 9.

### 3.2. Polymer Surface Modification

#### 3.2.1. Polyethylene Sample Preparation

Flat polyethylene (PE) samples with a density of 0.94 g/cm^3^ and a thickness of 2.0 mm were cut into rectangular shapes measuring 20.0 × 10.0 mm and 40.0 × 10.0 mm: the larger ones were used for WCA characterization, while the smaller ones were used for the other measurements. The samples were first cleaned for 20 min in an ultrasonic bath containing distilled water to remove any surface contaminants. Subsequently, the samples were rinsed in isopropanol for 20 min to ensure thorough cleaning. After cleaning, the samples were dried in an oven set at room temperature. Finally, the dried samples were treated with the APD for up to 30 min to prepare them for further analysis.

Polyethylene is the largest-volume polymer out of all polymers produced globally, and is known for its durability, flexibility, and chemical resistance. Its versatility and low cost make it an essential material in industries such as construction, healthcare, and consumer goods [85,86,87].

#### 3.2.2. Characterization of Polyethylene Samples

The wettability of the samples was assessed using a goniometer (Ramé-Hart 300 F1, Ramé, Succasunna, NJ, USA). Deionized water droplets (1.0 µL) were deposited along the longer axis of each sample, spaced 5.0 mm apart. Nine samples were measured for each treatment time, with both faces of the samples being evaluated. The mean water contact angle (WCA) of the droplets on all samples was calculated and plotted to assess the dependence on treatment time and the variation between the two faces of the PE samples. Changes in the molecular structure of the PE samples were analyzed using Fourier-transform infrared spectroscopy (FTIR) coupled with attenuated total reflectance (Perkin Elmer Spectrum 100 FTIR spectrometer, PerkinElmer, Shelton, CT, USA). Surface composition was examined using X-ray photoelectron spectroscopy (XPS) (Kratos AXIS Ultra instrument, Kratos, Manchester, UK). The surface was scanned along its longest symmetry axis, focusing on detecting carbon, oxygen, and nitrogen. The chemical composition of each sample was analyzed to determine the homogeneity of the treatment. XPS data provided insights into the uniformity and effectiveness of the treatment across the entire sample surface.

### 3.3. Microbial Inactivation

#### 3.3.1. Cytotoxicity Assay

**Figure 10:**
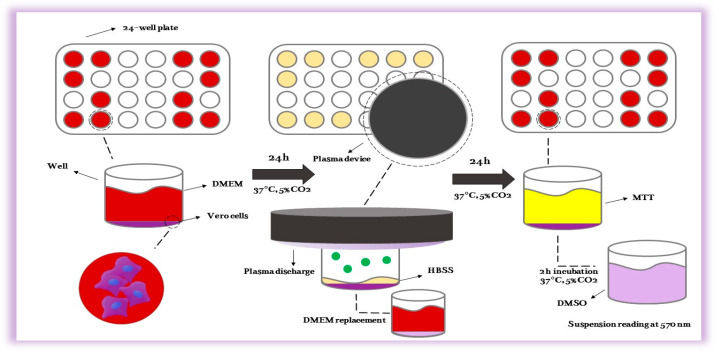
The scheme of the cytotoxicity test using the new plasma device in a 24-well plate.

Before any microbiological testing, all components underwent sterilization using UV light exposure, with exposure for 10 min. Subsequently, a cytotoxicity test was conducted to assess the impact of the APD on Vero cells. Vero cells were cultured with the utmost care at 37 °C in Dulbecco’s Modified Eagle Medium (DMEM), supplemented with 10% inactivated fetal bovine serum, 100 IU mL^−1^ of penicillin, and 100 µg.mL^−1^ of streptomycin, in an atmosphere of 95% air and 5% CO_2_. The cells were seeded at a density of 4.10^4^ cells/well in a 24-well plate, as illustrated in Figure 3, and incubated for 24 h at 37 °C [88]. After this initial incubation, the culture medium was removed, and 200 µL of Hank’s Balanced Salt Solution at pH 7.4 (HBSS) was added to each well. Subsequently, the cells underwent plasma exposure for 1, 3, 5, and 10 min, as outlined in Figure 10, while cells in HBSS without plasma exposure served as the control group.

**Figure 11:**
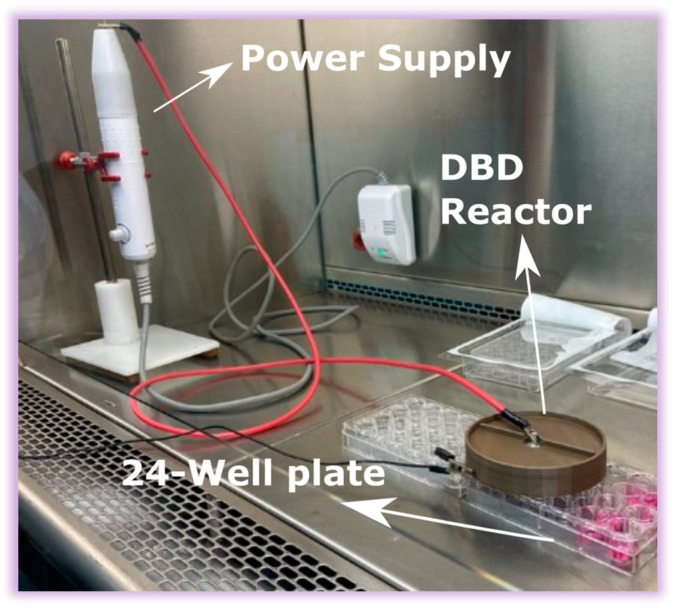
Exposure of Vero cells in a 24-well plate to the plasma generated by the affordable plasma device.

Twenty-four hours after the exposure to plasma (Figure 11), cell viability was assessed using the MTT (3-(4,5-dimethylthiazol-2-yl)-2,5-diphenyl tetrazolium bromide) assay according to ISO 10993-5/2009 standards [89]. Specifically, 500 µL of fresh PBS solution containing 0.5 mg/mL MTT was added to each well, followed by a three-hour incubation period. Subsequently, the MTT solution was replaced with DMSO to dissolve the formazan crystals, and the mixture was agitated for 15 min to ensure complete dissolution. The optical density of each well was then measured at 570 nm using a microplate reader. The absorbance data were normalized to the control group, set at 100%. All experiments were duplicated, with each condition being tested in 12 wells (n = 12). Several controls were included in the experimental design to ensure accuracy and reproducibility. Negative controls (cells without plasma exposure) and positive controls (cells treated with a known cytotoxic agent) were used to validate the assay’s performance. Additionally, the stability and consistency of the MTT reagent were verified by plotting a standard curve with known concentrations of a formazan solution. The results demonstrated a linear relationship between formazan concentration and optical density, confirming the reliability of the MTT assay under the specified conditions. The plasma exposure experiment aimed to evaluate potential cytotoxic effects on the treated cells, providing insights into the biocompatibility and safety of the plasma treatment. The normalized absorbance values were statistically analyzed to determine significant differences between treated and control groups, utilizing appropriate statistical methods such as ANOVAs followed by post hoc tests. These analyses ensured that the observed effects were statistically robust and biologically relevant.

#### 3.3.2. Antimicrobial Activity Assay

To investigate the antimicrobial efficacy, two clinically relevant microbial species were chosen: *Candida albicans*, a fungus responsible for both superficial and systemic human infections, and *Staphylococcus aureus*, a Gram-positive bacterium associated with various types of infections. The antimicrobial activity test followed a previously established methodology. Before the test, all components underwent UV light exposure within a laminar-flow hood for 10 min to ensure sterilization. Twenty-four-hour cultures of *C. albicans* (ATCC 18804) and *S. aureus* (ATCC 6538) were utilized. A standardized suspension containing 10⁶ cells/mL was prepared in sterile saline solution (0.9% NaCl) based on the spectrophotometric parameters outlined in Table 2. Subsequently, the suspension was spread on Sabouraud dextrose agar for *C. albicans* and Brain Heart Infusion (BHI) agar for *S. aureus* using a sterile Drigalski loop in a 100 µL volume. Each plate maintained a standardized agar volume of 15 mL. Once the suspension had dried on the agar plates, plasma treatment was administered for various durations: 1 min, 3 min, 5 min, and 10 min. Control plates were prepared without plasma exposure. The agar plates were then incubated at 37 °C for 24 h under aerobic conditions. All experiments were conducted in triplicate, and the results were recorded/are expressed as a percentage of reduction (%R). The %R was calculated using Equation (1), wherein []_F_ represents the colony-forming units per milliliter 24 h after treatment, and []_C_ denotes the control value of CFU/mL. The []_C_ value used for result calculation was the same concentration employed in preparing the inoculum, which was plated on agar before the test (1 × 10⁶ CFU/mL).

To further validate the results, the following additional controls were included:

(i) Negative controls: Plates with microbial cultures but no plasma treatment were included to assess the natural growth of *C. albicans* and *S. aureus*.
(ii) Positive controls: Plates treated with a known antimicrobial agent were included to assess the efficacy of the plasma treatment.

Statistical analyses were performed to determine significant differences between treated and control groups. The data were analyzed using ANOVAs followed by post hoc tests to ensure the reliability and reproducibility of the results.

The plasma treatment’s antimicrobial effect was visually confirmed by observing the reduction in colony formation on the agar plates. Additionally, the plates were photographed and digitally analyzed to quantify the reduction in microbial growth. This comprehensive approach provided robust data on the plasma treatment’s effectiveness against both *C. albicans* and *S. aureus*, contributing to understanding plasma’s potential as an antimicrobial agent.

(1) $$\%R=100-\frac{\left({\left[\;\right]}_{F}\times 100\right)}{{\left[\;\right]}_{C}}$$

## 4. Conclusions

In this work, an affordable DBD plasma device was developed and tested for surface modification and decontamination purposes. The device was manufactured using 3D printing and powered by a commercially available high-voltage power supply. By employing a metallic mesh as the grounded electrode, the species generated inside the reactor could freely flow through it and reach the surface without gas flow. The gas temperature remained close to room temperature regardless of the discharge power, making the device suitable for treating and decontaminating thermo-sensitive surfaces. Surface modification tests on polymers revealed slight changes with the insertion of O-containing functional groups and N species. These modifications could enhance a material’s adhesive properties and compatibility with other materials, making plasma-treated polymers more versatile for various applications. This potential to revolutionize the field of materials science and polymer chemistry is genuinely inspiring. Cell viability tests provided reassuring results. The plasma treatment was non-cytotoxic, with viability rates exceeding 90% for treatments up to 10 min. These results and the gas temperature findings suggest that in vivo tests might be feasible. This safety profile indicates potential biomedical applications for this device, such as wound healing and sterilization, instilling confidence in the device’s medical potential. Additionally, the device proved efficient in inhibiting *C. albicans* and *S. aureus*, primarily due to its effectiveness in generating reactive oxygen species (ROS). This ability to produce high amounts of ROS, including O_3_ and OH, highlights its potential for microbial inactivation and broad-spectrum antimicrobial applications. This plasma device has some advantages over other decontamination techniques, such as autoclaving, as autoclaving is a more expensive and time-consuming process, and some materials are too delicate for this method or involve surfaces that cannot be moved. Overall, the DBD plasma device proposed here presents an optimistic outlook. Its affordability, combined with its effectiveness and safety, positions it as an exciting candidate for further studies in the field of plasma technology. Its potential applications in materials science, healthcare, and beyond make it a promising tool for the future, offering hope for its widespread adoption and use.

## Figures and Tables

**Table 1:** Percentage reduction in *Candida albicans* and *Staphylococcus aureus* cell counts after treatment with the plasma device. The 1 × 10^6^ CFU/mL concentration was used for the control, which is the same as that used for the inoculum-plated samples.

Percentage Reduction (%)				
	1 Min	3 Min	5 Min	10 Min
*C. albicans*	99.992 ± 0.002	99.995 ± 0.003	99.997 ± 0.001	99.998 ± 0.001
*S. aureus*	99.980 ± 0.001	99.987 ± 0.003	99.985 ± 0.001	99.995 ± 0.001

**Table 2:** Information about the optical density (O.D.) and wavelength (*λ*) used for the preparation of each microorganism’s standardized inoculum in saline solution.

Microorganism	Wavelength (λ)	Optical Density (O.D.)
*S. aureus*	490	0.029
*C. albicans*	530	0.138

## Data Availability

The data that support the findings of this study are available from the corresponding author upon reasonable request.
